# A_2A_ Receptor Homodimer-Disrupting Sequence Efficiently Delivered by a Protease-Resistant, Cyclic CPP Vector

**DOI:** 10.3390/ijms20194937

**Published:** 2019-10-05

**Authors:** Maria Gallo, Gemma Navarro, Rafael Franco, David Andreu

**Affiliations:** 1 Department of Experimental and Health Sciences, Pompeu Fabra University, 08003 Barcelona, Spain; maria.gallo@upf.edu; 2 Department of Biochemistry and Physiology, University of Barcelona, 08028 Barcelona, Spain; dimartts@hotmail.com; 3 Centro de Investigación en Red Enfermedades Neurodegenerativas (CIBERNED), Instituto de Salut Carlos III, 28029 Madrid, Spain; 4 Faculty of Chemistry, University of Barcelona, 08028 Barcelona, Spain

**Keywords:** GPCR, adenosine receptors, homodimerization, GPCR function, disrupting peptides, trypsin

## Abstract

G-protein-coupled receptors associate into dimers/oligomers whose function is not well understood. One approach to investigate this issue is to challenge oligomerization by peptides replicating transmembrane domains and to study their effect on receptor functionality. The disruptor peptides are typically delivered by means of cell-penetrating vectors such as the human immunodeficiency virus (HIV) transcription trans-activation protein Tat. In this paper we report a cyclic, Tat-like peptide that significantly improves its linear analogue in targeting interreceptor sequences in the transmembrane space. The same cyclic Tat-like vector fused to a transmembrane region not involved in receptor oligomerization was totally ineffective. Besides higher efficacy, the cyclic version has enhanced proteolytic stability, as shown by trypsin digestion experiments.

## 1. Introduction

G-protein-coupled receptors (GPCRs), the most populated gene family in the human genome, are cell-surface receptors also known as heptaspanning receptors because they contain seven transmembrane (TM) alpha-helical domains. The widely used Kolakowski system groups GPCRs into six classes (A to F), with subclasses annotated using Roman numerals [1,2]. In addition to the seven TM helices, GPCRs also have intracellular C-terminal and extracellular N-terminal domains of variable length. Class C GCPRs (e.g., taste, glutamate or γ-aminobutyric acid receptors) are able to form a variety of homo and heteromers (reported as dimers or tetramers) when expressed in heterologous systems. These assemblies are consistent with 3D structural data showing that extracellular domains arrange into dimers [3,4,5,6,7,8]. Class A (rhodopsin-like) receptors, the most abundant GPCR group, was assumed for years to act as monomers, but this view has radically changed as many class A GPCRs are expressed as homo- and/or heteroreceptor complexes [9], and there is even in vivo evidence obtained by functional rescue using defective mutants of the gonadotropin releasing hormone receptor [10]. There are already several 3D structures for class A receptors and in many of the crystals the packing of molecules could fit with dimer formation in a biological membrane [11]. Recently the first 3D structure of a dimer of a full-length class C receptor, i.e., also containing the seven TM helical domains, has been reported for both inactive and active states [12]; this fact raises hopes for similar data coming soon for dimers of class A receptors.

One of the challenges in GPCR homo and heteromer research is to characterize the specific properties of the complexes, including differential functionality, if any. While potent tools exist to detect GPCR complex formation [9,13,14], the assessment of their functionality is, by far, more problematic. We have successfully shown, using chimeric peptide constructs, how disruption of homo/heteromer structure alters agonist-induced functionality and provides knowledge on the physiological role of GPCR receptor–receptor interactions. The most used peptide chimeras consist of the cell-penetrating peptide (CPP) sequence of the human immunodeficiency virus (HIV-1) trans-activation of transcription (Tat) protein, fused to a suitably chosen TM domain of the GPCR. Insertion into the membrane lipid bilayer and placement of such Tat-TM chimeras within the interaction interface alters the overall quaternary structure and can in turn assess the loss of function brought about by peptide-driven homo/heteromer disruption [15,16]. We have previously used this approach to decipher the structure and function of the complex formed by two class A adenosine GCPRs [17,18], and for other (adenosine-receptor-unrelated) heteroreceptor complexes [19,20].

As they crisscross from extracellular to intracellular space, TM helical domains adopt alternating orientations (i.e., N- to C-terminal in TM1, TM3, TM5 and TM7, C- to N-terminal in TM2, TM4 and TM6). This fact must be accounted for in the design of Tat-TM chimeras, to ensure that their insertion into cell membranes reproduces the native alignment. For instance, an interfering peptide based on the TM3 helix needs to be synthesized with the CPP moiety at the C-terminus, so that upon insertion the N-terminus of TM3 sequence will end up close to the extracellular space, and so on [21].

Despite the potential of the Tat-TM approach to modulate GPCR complex formation, experience indicates that the quantitative effect of linear Tat-TM constructs is limited. This can be attributed to transient, unproductive interactions causing “dilution” of the TM moiety within the membrane, but it also stems from the intrinsic lability of the Tat sequence, rich in basic (Arg, Lys) residues and, therefore, susceptible to the action of trypsin-like proteases or convertases [22,23]. Proteolytic degradation, a major hurdle in peptide therapeutic development, can be circumvented or at least mitigated in various ways; for instance, cyclization can protect the N- and/or C-termini from the action of exoproteases [24]. This strategy underlies some recent accounts of cyclic, Tat-inspired CPPs [25]. In tune with these findings, here we aim to compare the relative efficacy of cyclic vs. linear Tat-based CPPs in interfering with GCPR heteromer formation and function. We also show how our approach, where cyclization is combined with d-amino acid modification, also bears favorably upon resistance to proteolytic cleavage of the cyclic CPP construction.

## 2. Results and Discussion

### 2.1. Peptide Design and Synthesis

**Figure 1:**
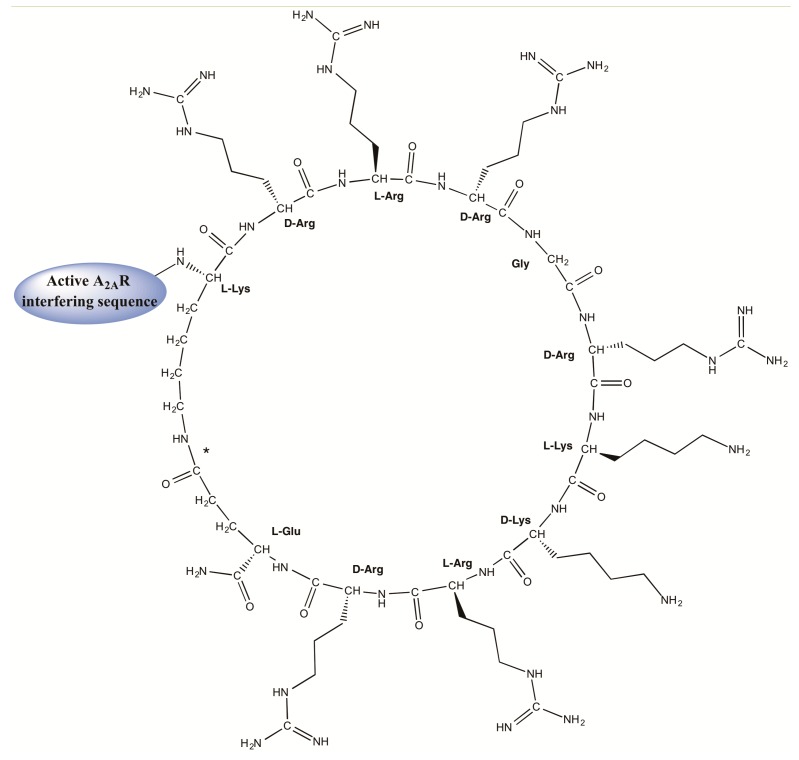
Structure of cyclic Tat-like construct KrRrGrKkRrE fused to A_2A_R transmembrane (TM)-interfering sequences. The amide bond between the ε–amino group of the N-terminal Lys and the γ–carboxyl of C-terminal Glu is marked with an asterisk.

This study uses two fully linear sequences (Table 1, entries **1** and **3**) and two peptides (entries **2** and **4**) where the TM5 and TM7 helical domains of A_2A_R are fused to a cyclic motif (Figure 1). Of the two TM domains, TM5 is a putative GPCR homodimer disruptor, whereas TM7 is not present in homodimer interfaces and thus often used as a negative control [17,18]. In peptides **2** and **4**, the cyclic moiety is generated by an intramolecular (amide bond) cyclization between the side chains of the N-terminal Lys (K) and C-terminal Glu (E) residues of the KrRrGrKkRrE sequence, inspired on the Tat(47–57) CPP sequence (YGRKKRRQRRR), and containing alternating l- and d- residues. All four peptides were efficiently obtained by state-of-the-art Fmoc solid-phase synthesis, with careful monitoring of the selective deprotection and cyclization steps of **2** and **4** (see Section 3.1 below). Further details are given in Table 1.

### 2.2. Effect of Transmembrane (TM)-Cyclic Cell-Penetrating Peptides (CPPs) as Disruptors of A_2A_ Receptor Dimerization

**Figure 2:**
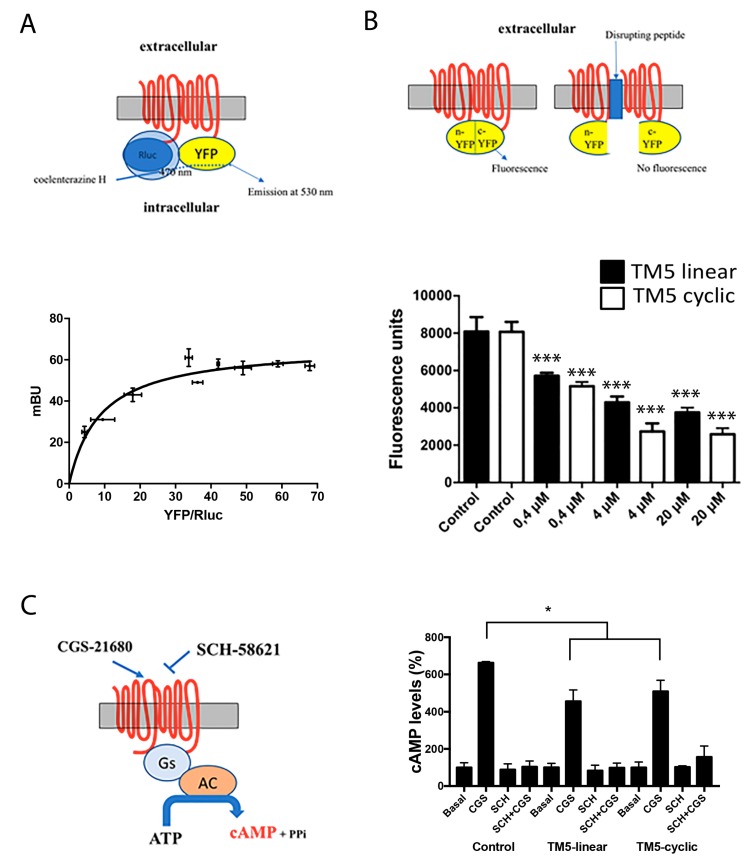
Bioluminescence resonance energy transfer (BRET) assays for homodimer receptor identification and bimolecular fluorescence complementation and signaling in cells expressing A_2A_ receptors in the presence of linear and cyclic Tat-like (disrupting) peptides **1** (linear TM5-Tat) and **2** (TM5-cyclic Tat-like). A scheme of BRET (**A**), molecular complementation (**B**) and cAMP determination (**C**) assays is shown close to each graph; AC = adenylyl cyclase. Panel A: data are mean +/− standard deviation (SD, *n* = 6 in duplicates). The blue arrow in the upper illustration denotes that, only if dimerization occurs, energy transfer from RLuc to coelenterazine H takes place, causing YFP excitation hence fluorescence emission. Panel B: data are mean +/− SD (*n* = 12 in duplicates); *** *p* < 0.001 respect to each control (analysis of variance (ANOVA) followed by Bonferroni’s post-hoc tests). Panel C: data are the mean (in % of increase over basal) +/- SD (*n* = 8 in duplicates). * *p* < 0.05 with respect to absence of peptides and no significant differences in linear versus cyclic by two-factor ANOVA analysis.

An heterologous system consisting of HEK-293T cells was used to express the adenosine A_2A_ receptor, which in these cells is able to form homodimers [26], as confirmed by bioluminescence resonance energy transfer (BRET) using cells transfected with cDNA for A_2A_Rluc (0.2 µg), increasing amounts of cDNA coding for A_2A_R-yellow fluorescent protein, and addition of the *Renilla* luciferase (Rluc) substrate, coelenterazine H. The saturable BRET curve displayed in Figure 2A proves molecular interaction between two molecules of the A_2A_R. These results on receptor homodimerization confirm those reported elsewhere [26].

For bimolecular fluorescence complementation (BiFC) assays: cells were transfected using cDNAs coding for two versions of the receptor. As described elsewhere [18,27], two cDNAs were prepared: each encoding the receptor fused to one of the two halves of the super yellow fluorescent protein (sYFP). 48 h post-transfection the fluorescence was determined in the absence and in the presence of linear peptides **1** and **3** or the corresponding cyclic peptides **2** and **4** after 4 h incubation. Each half of the fluorescent protein is unable, by itself, to emit fluorescence, which is only feasible after complementation of the two halves of the sYFP. If the peptide affects the structure of the dimer, the proteins are separated (to a greater or lesser extent) and fluorescence is reduced or lost. A comparison of the effect of the peptides on the “control” fluorescence, that is, the fluorescence given by the dimer in the absence of peptides is shown in Figure 2B.

TM7-displaying constructions, either linear (**3**) or cyclic (**4**), did not significantly affect the fluorescence of the control, thus confirming that TM7 does not participate in the dimerization (not shown). In contrast, both TM5-bearing peptides **1** and **2** affected the fluorescence of the control in a dose-dependent manner (Figure 2B). Interestingly, while the experiment shows linear peptide **1** perturbing A_2A_R dimerization, the cyclic version **2** produced a more marked, statistically significant, effect than its linear counterpart. *IC_50_* values were 0.52 µM and 0.35 µM for, respectively, the linear and the cycle-bearing molecules.

How linear and cyclic TM5 peptides affect signaling was addressed by determining cytosolic cyclic adenylic acid (cAMP) levels. As the A_2A_ receptor is coupled to Gs, its activation leads to increased cytosolic cAMP concentration. Both peptides significantly reduced the agonist (CGS-21680)-induced effect although with no statistical significance between data from linear and cyclic peptide (Figure 2). A control using the selective antagonist SCH-58621 blocked cAMP production under all conditions. The reduction in Gs-mediated signaling is moderate, in contrast with results with interfering (linear) peptides altering the quaternary structure and, subsequently, the signaling properties of GPCR heteromers. This difference may be explained by assuming two different GPCRs arranging into a heteromer made of two homodimers (stoichiometry 2:2) that can accommodate two different G proteins [17,18]. In this quaternary arrangement, interfering peptides for homodimers may be able to affect G coupling but only mildly, whereas the potency of interfering peptides for heteromers may be higher as they affect allosteric interactions occurring between the two different G proteins when the receptors are activated. In our experience, interfering peptides interacting with heteromeric interfaces have always been able to markedly alter the properties of the GPCR heteromer.

### 2.3. Peptide Susceptibility to Trypsin

**Figure 3:**
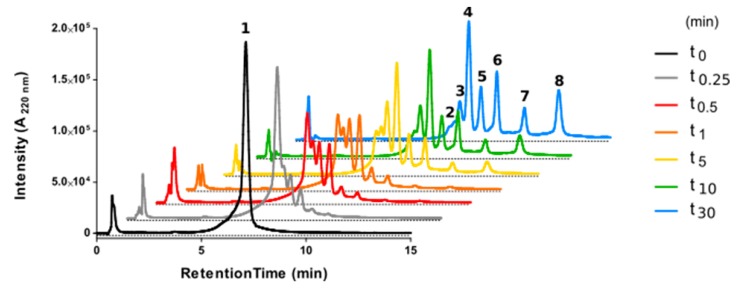
High-performance liquid chromatography (HPLC) analysis of fully linear peptide **1** on incubation with trypsin over 30 min. Starting material corresponds to peak **1** (black trace). Main tryptic fragments (**2**–**8**) are identified by mass on Table 2.

**Figure 4:**
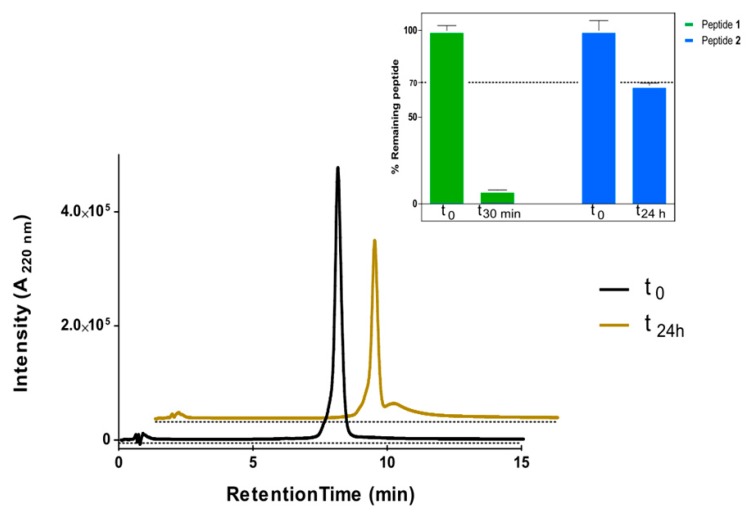
HPLC analysis of cyclic cell-penetrating peptides (CPP)-containing peptide **2** upon incubation with trypsin after 24 h. Inset: comparison of linear (**1**) and cyclic (**2**) peptide survival. Area of starting peak remaining at the stated time points is shown. Assays were done in duplicate. GraphPad prism was used to fit data.

It was of particular interest to ascertain how the internal cyclization and d-amino acid modifications influenced the lifespan of the partially cyclic structures. To this end, peptides **1** and **2,** both with a TM5 disruptor sequence fused to a linear or a cyclic CPP motif, respectively, were inspected by high-performance liquid chromatography (HPLC) upon trypsin treatment. Analysis confirmed the vulnerability of fully linear **1** (Figure 3), with a 90% decrease in the area of the starting peak after only 30 min (Figure 4), and the appearance of new peaks identified by mass spectrometry (MS) (Table 2). In sharp contrast, cyclic analog **2** proved highly impervious to digestion, with over 50% of the starting peptide unaltered after 24 h (Figure 4).

## 3. Materials and Methods 

### 3.1. Peptides

The sequences on Table 1 were assembled on 50 µmol (0.106 g; 0.47 mmol/g) of Fmoc-Rink-amide ChemMatrix resin in a Prelude instrument (Gyros Protein Technologies, Tucson, AZ) running optimized Fmoc synthesis protocols. Couplings with a 5-fold excess of Fmoc-amino acid, in the presence of 2-(1H-benzotriazol-1-yl)-1,1,3,3 tetramethyluronium hexafluorophosphate (HBTU, 5-fold excess) and N,N-diisopropylethylamine (DIEA, 10-fold excess), with N,N- dimethylformamide (DMF) as solvent for 5 + 5 min, were systematically repeated for all residues. Fmoc removal was done with 20% (*v*/*v*) piperidine in DMF for 2 × 2.5 min. Coupling and deprotection steps were separated by DMF washes (6 × 30 s). For the two fully linear sequences (Table 1), side chain protections were Boc (Lys, Trp), 2,2,4,6,7 pentamethyldihydrobenzofuran-5-sulfonyl (Arg), t-butyl (Glu, Ser, Thr, Tyr) and trityl (Asn, Cys, Gln, His). 

For sequences **2** and **4** containing the cyclic CPP motif (Figure 1), the Lys and Glu residues involved in intramolecular cyclization (Table 1) were orthogonally protected with (4-methoxyphenyl)diphenylmethyl (Mmt) and 2-phenyisopropyl (O-2-PhiPr) groups, respectively. In those syntheses, the C-terminal Fmoc-Glu (O-2-PhiPr)-OH (97.5 mg, 200 µmol) was loaded manually in the presence of HBTU (75.8 mg, 200 µmol) and DIEA (70 µL, 400 µmol), 1 h in DMF (double coupling). At the end of these two syntheses, the Fmoc group was removed and the N-terminal was capped by acetylation with acetic anhydride (37.81 µL, 400 µmol) and DIEA (140 µL, 800 µmol) in DMF for 1 h. After assembly, the peptide resins were treated with 1% (*v*/*v*) trifluoroacetic acid (TFA) in CH_2_Cl_2_ (4 × 10 min) for simultaneous orthogonal removal of O-2-PhiPr and Mmt groups, followed by neutralization with 5% DIEA in CH_2_Cl_2_ (4 × 5 min). Cyclization was next done with PyBOP/HOBt/DIPEA (5, 5, and 10 equiv) in DMF for 3 h, when a negative ninhydrin test was observed. A mini-cleavage with ~5 mg resin was also performed to check the purity and identity of the resin-bound product after the cyclization step.

Finally, all peptide-resins were treated with TFA/H_2_O/DODT/triisopropylsilane (94:2.5:2.5:1 *v*/*v*, 90 min) for full deprotection and cleavage. Peptides were isolated by precipitation with chilled diethyl ether, centrifuged for 3 × 10 min at 4 °C, taken up in H_2_O and lyophilized.

### 3.2. Peptide Analysis and Purification

Reconstitution of the lyophilized peptides was usually straightforward (H_2_O, acidified with HOAc if necessary). The crude peptides were analyzed by HPLC on Luna C_18_ columns (4.6 × 50 mm, 3 µm, Phenomenex, Torrance, CA, USA) in a LC-20AD system (Shimadzu, Kyoto, Japan). Elution was with linear gradients of solvent B (0.036% *v*/*v* TFA in CH_3_CN) into A (0.045% *v*/*v* TFA in H_2_O) over 15 min. Peptides were purified by preparative HPLC on Luna C_18_ columns (10 × 250 mm, 10 µm, Phenomenex) in a Shimadzu LC-8A instrument using linear gradients of solvent B (0.1% *v*/*v* TFA in CH_3_CN) into A (0.1% *v*/*v* TFA in H_2_O) over 30 min, at 7 mL/min flow rate with UV detection at 220 nm. Fractions of satisfactory purity (>95%) by analytical HPLC were pooled, lyophilized and analyzed for identity by HPLC-MS (C_18_ column, 4.6 × 150 mm, 3.5 µm, Phenomenex, Torrance, CA, USA) in a Shimadzu LC-MS 2010EV instrument, eluting with linear gradients of solvent B (0.08% *v*/*v* HCOOH in CH_3_CN) into A (0.1% *v*/*v* TFA in H_2_O) over 15 min at 1 mL/min flow rate.

### 3.3. Trypsin Digestion

A 1000 µg/1000 µL trypsin solution (Thermo Fisher Scientific, Cornellà de Llobregat, Spain) city, state abbreviation if available, country) was prepared by suspending the lyophilized enzyme in 50 mM ammonium bicarbonate buffer, pH 8.2. Peptides dissolved in the same buffer (1 µg/µL) were incubated with trypsin (peptide:trypsin ratio = 100:1) for 24 h at 37 °C. Samples were collected at various time points and digestion was stopped by addition of 20% (*v*/*v*) of 0.5% formic acid in H_2_O. Samples were stored at −20 °C until analyzed by reverse-phase HPLC-MS.

### 3.4. Bioluminescence Resonance Energy Transfer (BRET) and Bimolecular Fluorescence Complementation (BiFC) Assays

HEK-293T cells (American Type Culture Collection, ATCC, Mannassas, VA, USA) were transfected with a constant amount of cDNA for A_2A_Rluc (0.2 µg) (intramural production, see [17,18,26,28]) and increasing amounts of cDNA for A_2A_RYFP (0.1 to 0.5 µg) (intramural production, see [17,18,26,28]); 48 h after transfection cells were adjusted to 20 µg of protein using a Bradford assay kit (Bio-Rad, Munich, Germany) using bovine serum albumin for standardization. To quantify protein-YFP expression, fluorescence was read in a Mithras LB 940 multimode microplate reader (Berthold Technologies, Bad Wilbad, Germany) which allows the integration of the signals detected in the short-wavelength filter at 485 nm and the long-wavelength filter at 530 nm. For BRET measurements, readings were collected 1 min after addition of 5 µM coelenterazine H (Molecular Probes, Eugene, OR). To quantify protein-Rluc expression, luminescence readings were done 10 min after 5 µM coelenterazine H addition. Net BRET is defined as [(long-wavelength emission)/(short-wavelength emission)]–Cf, where Cf corresponds to [(long-wavelength emission)/(short-wavelength emission)] for the donor construct expressed alone in the same experiment. BRET is expressed as milli BRET, mBU (net BRET× 1,000) units. Data fitting with GraphPad Prism (San Diego, CA, USA) gave a saturation curve (Figure 2A) confirming that A_2A_R homodimers were formed under our experimental conditions.

To address homodimer disruption, bimolecular fluorescence complementation assays were performed using HEK-293T cells transfected with equal amounts of cDNAs (0.75 µg cDNA each) for A_2A_-cYFP and A_2A_-nYFP fusion proteins (intramural production, see [17,18,26,28]). In the absence of peptides, molecular complementation took place and YFP was reconstituted by homodimer formation (Figure 2B); accordingly, YFP fluorescence was readily detectable. Fluorescence was recorded in a Fluostar Optima fluorimeter (BMG Labtechnologies, Offenburg, Germany). Alternatively, cells were then treated with 0.4 µM of either of the four (**1**–**4**) peptides 4 h prior to the complementation assay, performed in supplemented Dulbecco’s modified Eagle’s medium (DMEM) medium. Values of fluorescence obtained in the absence or presence of peptides are given as mean ± standard error of the mean (SEM) of 16 independent experiments in duplicates. In all cases, one-way ANOVA followed by Bonferroni multiple comparison post hoc test were used for statistics analysis (*** *p* < 0.001; versus control. ^&^ *p* < 0.05 (Figure 2B)).

### 3.5. Cyclic Adenylic Acid(cAMP) Determination

HEK-293T cell growth medium was replaced by serum-starved DMEM medium 2 h prior to the experiment. Next, cells were detached, suspended in growing medium containing 50 µM zardaverine and plated in 384-well microplates (2,500 cells/well), pretreated (15 min) with vehicle and stimulated with the receptor agonist (CGS21680, 15 min) before adding 0.5 µM forskolin or vehicle. Readings were performed after 1h incubation at 25 °C. Homogeneous time-resolved fluorescence energy transfer (HTRF) measures were performed using the Lance Ultra cAMP kit (PerkinElmer, Waltham, MA, USA). Fluorescence at 665 nm was analyzed on a PHERAstar Flagship microplate reader equipped with an HTRF optical module (BMG Lab technologies, Offenburg, Germany).

## 4. Conclusions

As of today, the best tools to decipher the physiological role of GPCR oligomers are peptides that disrupt the quaternary structure and allow exploration of the consequences of heteromer-related loss of function. The disrupting peptides are optimally delivered as chimeric constructs where a TM segment interfering with oligomerization is fused with a cell-penetrating sequence. In the present work, we show that the efficacy of linear peptide constructs of this type can be significantly improved by cyclization of their CPP segment. Thus, our TM5-interfering peptide **2** was able to disrupt A_2A_R homodimer formation to a larger extent than the fully linear version **1**. The effect is clearly specific, as neither cyclic nor linear peptides containing the TM7 sequence, which does not lie within the dimer interface, were effective. A further attractive asset of the cyclic CPP strategy used here is that not only it makes the N- or C-termini of the CPP moiety impervious to proteases, but the alternating l- and d-amino acid pattern used to favor cyclization confers additional resistance of **2** to proteolysis, as shown by the remarkable stability (ca. 80% unaltered after 24 h) to trypsin, unmatched by the linear version.

In conclusion, we believe our TM5-interfering peptide **2** provides proof-of-principle for the design of optimized peptide-based disruptors of GCPR functionality, opening new perspectives for in vivo studies on the differential expression of functional A_2A_R homodimers in the brain and their implications in central nervous system (CNS) disease.

## Figures and Tables

**Table 1:** Peptides used in this study.

Peptide	Description	Sequence	MW, Da
**1**	Linear Tat-based TM5	MNYMVYFNFFACVLVPLLLMLGVYLYGRKKRRQRRR-amide	4514.60
**2**	Cyclic Tat-like TM5 ^a^	MNYMVYFNFFACVLVPLLLMLGVYL[KrRrGrKkRrE]-amide	4504.60
**3**	Linear Tat-based TM7	LWLMYLAIVLSHTNSVVNPFIYAYYGRKKRRQRRR-amide	4369.22
**4**	Cyclic Tat-like TM7 ^a^	LWLMYLAIVLSHTNSVVNPFIYAY[KrRrGrKkRrE]-amide	4359.22

^a^ The sequence within brackets denotes intramolecular, amide bond cyclization between the Lys (K) and the C-terminal Glu (E) residues; N-terminus is acetylated.

**Table 2:** Primary structures and physicochemical properties of linear peptide **1** and its tryptic fragments (Figure 3).

Peak Number	Retention Time (min) ^a^	Amino Acid Sequence ^b^	Theoretical Mass (Da)	Experimental Mass (Da)
1	7.21	MNYMVYFNFFACVLVPLLLMLGVYLYGRKKRRQRRR-amide	4514.60	4514.00
2	7.46	MNYMVYFNFFACVLVPLLLMLGVYLYGRKKRRQRR-carboxyl	4359.40	4358.20
3	7.76	MNYMVYFNFFACVLVPLLLMLGVYLYGRKKRRQR-carboxyl	4203.20	4202.40
4	8.24	MNYMVYFNFFACVLVPLLLMLGVYLYGRKKRR-carboxyl	3918.89	3919.05
5	8.81	MNYMVYFNFFACVLVPLLLMLGVYLYGRKKR-carboxyl	3762.70	3763.35
6	9.57	MNYMVYFNFFACVLVPLLLMLGVYLYGRKK-carboxyl	3606.51	3606.45
7	10.90	MNYMVYFNFFACVLVPLLLMLGVYLYGRK-carboxyl	3478.34	3477.75
8	12.62	MNYMVYFNFFACVLVPLLLMLGVYLYGR-carboxyl	3350.16	3351.60

^a^ Peptides were analyzed by HPLC with a 40–90% linear gradient of acetonitrile over 15 min. Identity was established by HPLC-mass spectrometry (MS) (details in Materials and Methods); ^b^ Residues in red correspond to the cell-penetrating sequence.

## References

[1] Attwood T.K., Findlay J.B. (1994). Fingerprinting G-protein-coupled receptors. Protein Eng..

[2] Kolakowski L.F.J. (1994). GCRDb: A G-protein-coupled receptor database. Recept. Channels.

[3] George S.R., O’Dowd B.F., Lee S.P. (2002). G-protein-coupled receptor oligomerization and its potential for drug discovery. Nat. Rev. Drug Discov..

[4] Jacoby E., Bouhelal R., Gerspacher M., Seuwen K. (2006). The 7 TM G-protein-coupled receptor target family. Chem. Med.Chem..

[5] Reyes-Cruz G., Hu J., Goldsmith P.K., Steinbach P.J., Spiegel A.M. (2001). Human Ca2+ receptor extracellular domain. Analysis of function of lobe I loop deletion mutants. J. Biol. Chem..

[6] Remelli R., Robbins M.J., McIlhinney R.A.J. (2008). The C-terminus of the metabotropic glutamate receptor 1b regulates dimerization of the receptor. J. Neurochem..

[7] White J.H., Wise A., Main M.J., Green A., Fraser N.J., Disney G.H., Barnes A.A., Emson P., Foord S.M., Marshall F.H. (1998). Heterodimerization is required for the formation of a functional GABA(B) receptor. Nature.

[8] Kunishima N., Shimada Y., Tsuji Y., Sato T., Yamamoto M., Kumasaka T., Nakanishi S., Jingami H., Morikawa K. (2000). Structural basis of glutamate recognition by a dimeric metabotropic glutamate receptor. Nature.

[9] Franco R., Martinez-Pinilla E., Lanciego J.L., Navarro G. (2016). Basic Pharmacological and Structural Evidence for Class A G-Protein-Coupled Receptor Heteromerization. Front. Pharm..

[10] Rivero-Muller A., Chou Y.-Y., Ji I., Lajic S., Hanyaloglu A.C., Jonas K., Rahman N., Ji T.H., Huhtaniemi I. (2010). Rescue of defective G protein-coupled receptor function in vivo by intermolecular cooperation. Proc. Natl. Acad. Sci. USA.

[11] Cordomi A., Navarro G., Aymerich M.S., Franco R. (2015). Structures for G-Protein-Coupled Receptor Tetramers in Complex with G Proteins. Trends Biochem. Sci..

[12] Koehl A., Hu H., Feng D., Sun B., Zhang Y., Robertson M.J., Chu M., Kobilka T.S., Laeremans T., Steyaert J. (2019). Structural insights into the activation of metabotropic glutamate receptors. Nature.

[13] Franco R., Casadó V., Cortés A., Ferrada C., Mallol J., Woods A., Lluis C., Canela E.I., Ferré S. (2007). Basic concepts in G-protein-coupled receptor homo- and heterodimerization. Sci. World J..

[14] Ferre S., Baler R., Bouvier M., Caron M.G., Devi L.A., Durroux T., Fuxe K., George S.R., Javitch J.A., Lohse M.J. (2009). Building a new conceptual framework for receptor heteromers. Nat. Chem. Biol..

[15] Hasbi A., Perreault M.L., Shen M.Y.F., Zhang L., To R., Fan T., Nguyen T., Ji X., O’Dowd B.F., George S.R. (2014). A peptide targeting an interaction interface disrupts the dopamine D1-D2 receptor heteromer to block signaling and function in vitro and in vivo: Effective selective antagonism. Faseb J. Off. Publ. Fed. Am. Soc. Exp. Biol..

[16] Tripathi A., Vana P.G., Chavan T.S., Brueggemann L.I., Byron K.L., Tarasova N.I., Volkman B.F., Gaponenko V., Majetschak M. (2015). Heteromerization of chemokine (CXC motif) receptor 4 with α1A/B-adrenergic receptors controls α1-adrenergic receptor function. Proc. Natl. Acad. Sci. USA.

[17] Navarro G., Cordomí A., Brugarolas M., Moreno E., Aguinaga D., Pérez-Benito L., Ferre S., Cortés A., Casadó V., Mallol J. (2018). Cross-communication between Gi and Gs in a G-protein-coupled receptor heterotetramer guided by a receptor C-terminal domain. BMC Biol..

[18] Navarro G., Cordomí A., Zelman-Femiak M., Brugarolas M., Moreno E., Aguinaga D., Perez-Benito L., Cortés A., Casadó V., Mallol J. (2016). Quaternary structure of a G-protein-coupled receptor heterotetramer in complex with Gi and Gs. BMC Biol..

[19] Aguinaga D., Medrano M., Cordomí A., Jiménez-Rosés M., Angelats E., Casanovas M., Vega-Quiroga I., Canela E.I., Petrovic M., Gysling K. (2019). Cocaine blocks effects of hunger hormone, ghrelin, via interaction with neuronal sigma-1 receptors. Mol. Neurobiol..

[20] Navarro G., Quiroz C., Moreno-Delgado D., Sierakowiak A., McDowell K., Moreno E., Rea W., Cai N.-S., Aguinaga D., Howell L.A. (2015). Orexin-corticotropin-releasing factor receptor heteromers in the ventral tegmental area as targets for cocaine. J. Neurosci..

[21] Jastrzebska B., Chen Y., Orban T., Jin H., Hofmann L., Palczewski K. (2015). Disruption of Rhodopsin Dimerization with Synthetic Peptides Targeting an Interaction Interface. J. Biol. Chem..

[22] Antalis T.M., Buzza M.S., Hodge K.M., Hooper J.D., Netzel-Arnett S. (2010). The cutting edge: Membrane-anchored serine protease activities in the pericellular microenvironment. Biochem. J..

[23] Basak A. (2005). Inhibitors of proprotein convertases. J. Mol. Med..

[24] Yao J.-F., Yang H., Zhao Y.-Z., Xue M. (2018). Metabolism of Peptide Drugs and Strategies to Improve their Metabolic Stability. Curr. Drug Metab..

[25] Nischan N., Herce H.D., Natale F., Bohlke N., Budisa N., Cardoso M.C., Hackenberger C.P.R. (2015). Covalent attachment of cyclic TAT peptides to GFP results in protein delivery into live cells with immediate bioavailability. Angew. Chem. Int. Ed. Engl..

[26] Canals M., Burgueno J., Marcellino D., Cabello N., Canela E.I., Mallol J., Agnati L., Ferre S., Bouvier M., Fuxe K. (2004). Homodimerization of adenosine A2A receptors: Qualitative and quantitative assessment by fluorescence and bioluminescence energy transfer. J. Neurochem..

[27] Navarro G., Carriba P., Gandía J., Ciruela F., Casadó V., Cortés A., Mallol J., Canela E.I., Lluis C., Franco R. (2008). Detection of heteromers formed by cannabinoid CB1, dopamine D2, and adenosine A2A G-protein-coupled receptors by combining bimolecular fluorescence complementation and bioluminescence energy transfer. Science.

[28] Hinz S., Navarro G., Borroto-Escuela D., Seibt B.F., Ammon C., De Filippo E., Danish A., Lacher S.K., Červinková B., Rafehi M. (2018). Adenosine A2A receptor ligand recognition and signaling is blocked by A2B receptors. Oncotarget.

